# Early detection of median nerve compression by Electroneurography can improve outcome in children with Mucopolysaccharidoses

**DOI:** 10.1186/s13023-018-0937-9

**Published:** 2018-11-21

**Authors:** Kim Maincent, Bénédicte Héron, Thierry Billette de Villemeur, Michèle Mayer

**Affiliations:** 1 Hospital for Pediatrics and Reeducation, Bullion, 78830 France; 2 Department of Pediatric Neurology, CHU Trousseau, APHP, Paris, France; 3 Reference Center for Lysosomal Diseases, CHU Trousseau, APHP, Paris, France; 4 Reference Center for Neuromuscular diseases, CHU Trousseau, APHP, Paris, France; 5 Clinical Electrophysiology Laboratory, CHU Trousseau, APHP, Paris, France

**Keywords:** Mucopolysaccharidoses, Hunter, Hurler, Hurler-Scheie, Nerve conduction study, Electroneurography, Carpal tunnel syndrome, Median nerve compression

## Abstract

**Background:**

Carpal tunnel syndrome (CTS) is a common complication of the mucopolysaccharidoses. In severe or attenuated mucopolysaccharidoses patients, clinical symptoms of CTS usually appear at a late stage of median nerve compression. Relying on CTS symptoms is often too late and there is a risk of axonal damage and further irreversible sequelae. Electroneurography is a powerful technique to detect the initial preclinical signs of median nerve compression. In a retrospective series of 13 children with mucopolysaccharidoses (10 Hunter, one Hurler-Scheie and 2 Hurler children), we describe the electroneurography progression of CTS (43 hand evaluations) and the severity of median nerve damage.

**Results:**

The average age at mucopolysaccharidoses diagnosis was 33.6 months (11–66 months). Clinical signs of CTS appeared on average 44.6 months (0–73 months) after diagnosis of mucopolysaccharidoses. Electroneurography anomalies suggestive of CTS appeared as early as the age of 3.5 years and probably preceded clinical signs of CTS. Median nerve compression was bilateral and distal, initially on the sensory pathway then becoming motor-sensory. Beyond a threshold of 14 m/sec median distal motor nerve conduction velocity (MNCV_d_) and index of terminal latency (MNCVd/MNCVp) of 0.27, there was true distal conduction slowdown.

**Conclusions:**

To prevent irreversible sequelae of median nerve compression, we suggest annual electroneurography testing for mucopolysaccharidoses patients starting as early as 3 years of age, including both motor and sensory nerve pathways, on median and, in reference to the ulnar nerves, bilaterally at the wrist and the elbow. Timely surgical intervention can greatly improve the overall function and quality of life of these patients.

## Introduction

The mucopolysaccharidoses (MPS) are lysosomal storage disorders caused by defective catabolism of complex molecules, namely the glycosaminoglycans (GAG) and their consequent accumulation in cells and tissues. Their overall incidence is 1:25,000 to 1:30,000 births [1, 2]. Seven clinical subtypes have been described (MPS I-IV, VI, VII and IX) due to 11 enzyme deficiencies, coded by 11 different genes. MPS I (estimated incidence 1:100,000 births) and MPS II (estimated incidence 1:80,000 male births) are the more frequent. The MPS are chronic, progressive multivisceral disorders and show a large variability in age of onset, rate of progression and severity [3, 4]. Symptoms occur in early infancy or childhood in the most severe patients. The accumulation of mucopolysaccharides in the brain can lead to cognitive regression with loss of language and impairment of both gross and fine motor skills, as well as behavioral problems characterized by motor agitation and aggression. All cases have some degree of multiple dysostose bilaterally affecting hands, arms, legs, hips, and the spine. The resulting morphology of the hands is striking; finger joints become deformed, stiff and claw-like due to the inflexibility of the interphalangeal joints [5].

Carpal tunnel syndrome (CTS) is a common complication of the MPS I (Hurler, Hurler-Scheie, and Scheie), MPS II (Hunter), and MPS VI (Maroteaux-Lamy) syndromes. The combination of bone dysplasia and accumulation of surcharge products (GAG) in the connective tissue of tendons results in reduced space in the carpal canal. This osteo-ligament modification leads to the entrapment and compression of the median nerve [6–10].

In MPS I and MPS II, early initiation of therapies with hematopoietic stem cell transplantation and/or enzyme replacement therapy (ERT) improves outcome, and can prevent or delay the development of irreversible disease manifestations [11–14]. Although ERT is effective in decreasing GAG accumulation, this has not been shown to correlate with clinical improvement of CTS in the long run, and many children have required surgical interventions following ERT [15].

Early clinical signs and symptoms of CTS in children are usually nonspecific and differ from that seen in adults. Pain remains a frequent symptom; the child usually has frequent bouts of crying, especially at night, with hand rubbing. Delayed diagnosis is usually due to the subtle or atypical symptoms compounded by the often lack of communication skills due to young ages or intellectual disabilities secondary to MPS [8, 10, 14, 16]. CTS should be confirmed and managed by routine electroneurography (ENG) in MPS patients [17, 18], rendering the possibility of surgical decompression [19] to avoid significant and permanent loss of hand function [8, 20].

However, there are very few data in the literature describing ENG exploration of CTS in MPS children. Here we present a retrospective series of 13 children with MPS I and MPS II. We describe the severity of median nerve damage and the ENG evolution of CTS in these children. We also give recommendations for timely ENG screening and follow-up to guide the decision for surgical decompression.

## Materials and methods

### Patients

We retrospectively studied a series of 13 children with enzymatic or molecular confirmed MPS I or II, referred to the Reference Center for Lysosomal Diseases and who underwent ENG evaluations in the Laboratory of the Trousseau hospital in Paris, France, between 1996 and 2011.

The following information was collected from medical and clinical records: birth history, clinical signs and symptoms, age at MPS diagnosis, age at CTS symptoms onset, and therapies received (ERT or surgical decompression).

This retrospective study was a review of clinical and ENG data and did not require Ethics Committee approval according to French regulations. The parents of all the children gave their consent for the medical records to be used and all data processing was carried out in compliance with the French Information Technology and Privacy Law.

### Electroneurography evaluation

ENG evaluations were performed according to the Nerve Conduction Study (NCS) guidelines prepared by the Société de Neurophysiologie Clinique de Langue Française [21] using a Viking (Nicolet) until 2005, Spirit (Nicolet) until 2007 and a Viasys (FMB) thereafter. ENG studies were performed by the electrophysiologist and pediatric neurologist and the results interpreted blindly by the investigator before being collated with respective clinical chart review data. Age-adjusted normal calculation tables for the median and ulnar nerves as established by Mayer and Raimbault respectively were referenced to account for the degree of myelination at young ages [22]. Reference tables were required because nerve conduction velocity varies with age due to myelin maturation and latencies vary with the child’s stature.

Motor and sensory nerve conduction studies were done bilaterally using surface recordings and stimulations while maintaining skin temperature of the patients above 32 °C. It should be noted that examination conditions are often very difficult, especially in a frightened and agitated child who cannot express himself orally. These examinations require great patience and at the same time should be rapidly executed. The child lay on the knees of his parent without restraints. For the duration of each evaluation, the child was comforted by explaining each manipulation and each electrical stimulation.

### Recorded and calculated parameters

#### Motor nerve conduction

Median and ulnar motor nerves were stimulated at the wrist and at the elbow using bipolar surface electrodes. The stimulation was characterized by duration of 0.10 milliseconds and supramaximal intensity. Responses were collected downstream at the abductor pollicis brevis and adductor digiti minimi for stimulation of the median and ulnar nerves respectively.

For both the median and ulnar nerves, distal and proximal motor nerve measurements (Amplitude CMAP_d_,_p_ in millivolts, and Latency MDL in milliseconds) were recorded in response to stimulations at the wrist and elbow respectively. The motor nerve conduction velocity (MNCV) was calculated along this segment for both median and ulnar nerves (m/sec). Distal MNCV for both median and ulnar nerves was calculated as distal distance/distal latency, along the segment between the point of stimulation and that of recording. It should be noted that MNCV_d_ does not cover a homogeneous area (terminal nerve endings and neuromuscular junction). However, it is interesting to monitor as it represents distal conductivity independent of the variability related to the length of the hand and the height of the stimulation point at the wrist.

#### Sensory nerve conduction

The sensory nerve was stimulated orthodromically using two circular electrodes placed around the 2nd and 3rd fingers (for the median nerve) or around the 4th and 5th fingers (for the ulnar nerve), the active electrode (cathode) proximal to the wrist. Responses were collected distally and proximally on the corresponding nerve using surface electrodes at the wrist and elbow on the locations marked during the motor nerve conduction test. Each stimulation lasted 0.20 milliseconds. Between 10 and 50 evoked potentials were averaged to reliably confirm the presence or absence of a reading and to distinguish the response from background noise.

For both the median and ulnar nerves, distal and proximal sensory nerve measurements (latency SDL in milliseconds and amplitude of response SEPA_d_,_p_ in microvolts), were recorded after each stimulus. The velocity of sensory nerve conduction SNCV was calculated as distance over time of conduction (m/sec). The distal sensory velocities (SNCV) were calculated as mentioned above.

### Severity index

The following grading scheme for the severity of CTS by EDX criteria [23] was applied after each examination:

- *Mild CTS*—prolonged median SDL ± SEPA below the lower limit of normal (LLN).
- *Moderate CTS*— prolonged median SDL and prolongation of median MDL
- *Severe CTS*—prolonged median MDL and SDL, with either an absent or low SEPA or a diminution of CMAP.

## Results

### Patients

This series consisted of 13 children born between 1994 and 2005 (Table 1): 10 boys had Hunter, one boy had Hurler-Scheie, one boy and one girl had Hurler syndrome. Ten children presented with motor hyperactivity and severe cognitive deficit, with lack of language skills. One of the Hurler patients had moderate psychomotor delays at the time of diagnosis (18 months). The child with attenuated Hunter and the child with Hurler-Scheie had learning difficulties without behavioral problems.

**Table 1:** Electroneurography examination results

Case	Gender	Diagnosis	age at CTS surgery (months)	age at ERT (months)	Age at ENG (months)	*Distal Motor Latency MNL (msec)*		*Distal Sensory Latency SNL (msec)*		*Distal Motor Amplitude CMAP (mVlt)*		*Distal Sensory Amplitude SEPA (μVlt)*	
						right	left	right	left	right	left	right	left
N°1	M	Hunter	84	125	55	3.8 (+2.5 SD)	4.2 (+3 SD)	2.6 (+2 SD)	3.6 (+4 SD)	3.7 (77%)	3.0 (62%)	9.4 (47%)	8.7 (43%)
					70	4.7 (+3.5 SD)	5.8 (+6 SD)	3.2 (+3 SD)	absent	0.6 (12%)	1.1 (23%)	9.5 (47%)	absent
					79	4.5 (+3 SD)	6.6 (+7 SD)	2.7 (+2 SD)	absent	8.8 (183%)	6.2 (125%)	5.0 (22%)	absent
N°2	M	Hunter	141	145	146	5.0 (+3 SD)	3.5 (0 SD)	3.0 (+2 SD)	2.5 (0 SD)	NA	NA	NA	4.1 (0%)
					179	2.3 (-1 SD)	3.5 (+0.8 SD)	absent	absent	3.1 (76%)	3.2 (77% )	absent	absent
N°3	M	Hunter	136	141	142	6.7 (+6 SD)	6.0 (+5 SD)	3.7 (+4 SD)	3.2 (+4 SD)	4.5 (110%)	4.5 (110%)	4.7 (31%)	8.3 (56%)
					167	4.1 (+1.5 SD)	3.7 (+1 SD)	2.8 (+1.5 SD)	2.5 (+0.5 SD)	3.8 (93% )	3.9 (95%)	11.0 (74%)	23.3 (156%)
N°4	M	Hunter	78	35	31	normal	normal	normal	normal	normal	normal	normal	normal
					57	3.0 (+1 SD)	4.8 (+5 SD)	3.2 (+3 SD)	3.4 (+3.5 SD)	3.8 (80%)	5.3 (110%)	13.3 (66%)	14.0 (70%)
N°5	M	Hunter	78	109	69	13.2 (+18 SD)	10.0 (+13 SD)	absent	absent	0.6 (13%)	0.6 (15%)	absent	absent
					76	11.0 (+14 SD)	10.7 (+14 SD)	absent	absent	0.7 (14%)	0.2 (4%)	absent	absent
					102	5.3 (+3 SD)	3.7 (+1 SD)	4.2 (+7 SD)	3.8 (+6 SD)	2.5 (73%)	5.6 (164%)	4.9 (38%)	5.0 (38%)
N°6	M	Hunter	120	129	134	3.0 (0 SD)	4.0 (+1.5 SD)	3.4 (+3 SD)	3.12 (+2.5 SD)	4.9 (120%)	4.8 (117%)	1.9 (12%)	19.6 (130%)
N°7	M	Hunter		68	115	4.5 (+2 SD)	4.3 (+2 SD)	3.3 (+4 SD)	3.3 (+4 SD)	8.3 (265%)	2.5 (74%)	8.5 (65%)	5.0 (38%)
N°8	M	Hunter			72	3.7 (+3 SD)	3.5 (+2.5 SD)	absent	absent	0.6 (15%)	4.4 (110%)	absent	absent
N°9	M	Hunter			44	5.4 (+5 SD)	4.7 (+4 SD)	3.8 (+5 SD)	3.0 (+1 SD)	1.9 (48%)	0.5 (13%)	10.6 (53%)	8.4 (42%)
N°10	M	Hunter	53		43	4.2 (+3 SD)	4.9 (+7 SD)	2.4 (+1 SD)	3.8 (+5 SD)	4.3 (107%)	2.5 (62%)	25.0 (120%)	17.1 (85%)
N°11	M	Hurler			56	5.1 (+4.5 SD)	4.1 (+3 SD)	4.1 (+5 SD)	2.5 (+1.5 SD)	1.0 (20%)	2.9 (60%)	3.7 (18%)	12.8 (65%)
N°12	F	Hurler		12	18	2.4 (0 SD)	2.2 (0 SD)	128.0 (-2 SD)	1.1 (-2 SD)	7.2 (185%)	4.8 (125%)	29.6 (116%)	42.6 (167%)
N°13	M	Hurler-Sheie	74	93	51	2.5 (0 SD)	ND	absent	ND	5.2 (108%)	ND	absent	ND
					64	2.0 (+0.5 SD)	2.2 (0 SD)	absent	absent	2.3 (49%)	4.6 (95% )	absent	absent
					176	4.5 (+ 2.5 SD)	3.6 (+1 SD)	absent	absent	2.1 (51%)	5.9 (144%)	absent	absent

*CTS* carpal tunnel syndrome, *ND* not done, *NA* not available, *ERT* Enzyme Replacement Therapy, *ENG* electroneurography evaluation, *SD* standard deviation, *%* percent of lower limit of normal (LLN)

The average age at MPS diagnosis was 33.6 months (11–66 months). The average age at the first ENG evaluation was 6.25 years (median 56 months; 31–146). CTS symptoms onset dates (available for 5 cases) appeared on average at 78 months of age (43–109 months). Predominant symptoms reported by the parents were agitation and pain; withdrawing hands, crying when touched, or waking up during the night with hands rubbing against each other. Parents also reported increased difficulty with fine motor skills; inability to self-feed, hold a pencil, and difficulty manipulating a game remote. On first presentation, we noted the claw-like appearance of all the hands and stiffness of the fingers, which are very early symptoms preceding those of CTS.

A total of eight children underwent decompression surgery at an average age of 95.5 months (7.95 years). Six of these children had already received ERT and one received ERT just following surgery.

Nine children had received ERT at an average age of 7.9 years (12–145 months), two of whom did not have surgery (aged 12 and 68 months). Clinical improvement following infiltration was evidenced by finer hand features, increased joint flexibility and hand functionality.

### Electroneurography evaluation

All 13 children underwent bilateral sensory and motor ENG evaluations, generating 43 hand ENG recordings (Table 1). In one child it was not possible to get bilateral ENG recordings on the first ENG visit (#13a). Only one child had complementary needle electromyography, with normal results.

Two children had normal ENG results on their first exam and were clinically asymptomatic (# 3,4). Eleven other children showed ENG anomalies straight away on at least one hand, four of whom were clinically asymptomatic.

Steven’s severity grading scheme was applied to each of the 43 hand ENGs. A total of 15 exams were indicative of severe compression of the median nerve; 10 with moderate severity; 8 with minor severity; 10 were normal. For all exams with minor severity, we measured a prolonged SDL of + 3.5 SD to +7SD associated with decreased SEPA_d_ of 18% to 85% of LLN. For these with moderate severity, SDL was prolonged of + 2SD to +6SD associated with decreased SEPA_d_ of 22% to 66% of LLN, and MDL were prolonged + 2SD to +7DS.

Severe compression was marked by an absence of both SDL and SEPA_d_. MDL were prolonged +2.5SD to +18SD in 10 exams and normal in the rest. Distal CMAP was > 95% of LLN in 5 of the exams and < 100% (4–77%) of LLN in the rest.

Early signs of CTS were expressed first on sensory then progressively on both motor and sensory median nerve pathways. When both pathways were affected, the impact was more pronounced on the sensory as seen in 9 of 13 cases (#1–3, 5–8, 10, 13). The anomaly was generally bilateral, though in one patient the pathology was expressed predominantly on one side (#2).

Proximal MNCV were measured for 10 patients and were in the normal range bilaterally for 9 patients (#2–4, 6, 7, 9, 11–13). Amplitude ratios (proximal over distal) did not decrease. One patient (#5) had a particularly severe compression of the median nerve, with prolonged bilateral MDL (+14SD to +18SD), significantly reduced amplitudes (13% to 15% of LLN), and unsynchronized muscle responses. This was the only patient to display a discrete slowing of MNCV (+3SD and + 4SD) which probably indicates a retrograde degeneration of the nerve fibers that is inevitable in view of the severity of compression in the canal.

Index of terminal latency (ITL) is the ratio of MNCV_d_ to MNCV_p_. The ITL adjusts the median motor distal latency for the terminal conduction distance and the proximal nerve conduction velocity.

The relationship between distal latencies, conduction velocities MNCV and ITL in 41 hand ENG evaluations is presented in Table 2. In all recordings where MDL was greater than 2SD there was a corresponding MNCV_d_ < 13 m/s and when MNCV_p_ was measured, ITL < 0.26 on 14 hand ENG evaluations. Likewise, when MDL was less than 2SD, there was a corresponding MNCV_d_ > 14 m/s and ITL > 0.26.

**Table 2:** Relationship between the standard deviation (SD) of Motor Nerve Distal Latency (MNL_d_) and Index of Terminal latency (ITL) in 13 cases and 41 hand ENG evaluations

*SD* ^*¥*^ *of MNL*_*d*_	*MNCV*_*p*_ *(m/s)*	*MNCV*_*d*_ *(m/s)*	*ITL*	*Case*	*Hand*
0.0	55.0 (-0.5 SD)	20	0.36	6	Right
0.0	51.0 (+0.5 SD)	17	0.33	12	Right
0.0	53.0 (0 SD)	18	0.36	13a	Right
0.0	43.0 (-0.5 SD)	17	0.40	12	Left
0.0	*ND*	18		2a	Left
0.5	*ND*	25		13b	Right
0.5	*ND*	23		13b	Left
0.8	47.9 (-2 SD)	18	0.38	2b	Left
1.0	58.2 (0 SD)	29	0.50	2b	Right
1.0	54.3 (-0.5 SD)	17	0.31	4	Right
1.0	56.5 (0 SD)	16	0.28	3b	Left
1.0	46.9 (-2 SD)	15	0.32	5c	Left
1.0	52.0 (-1 SD)	17	0.33	13c	Left
1.5	55.9 (-0.5 SD)	15	0.27	3b	Right
1.5	55.0 (-0.5 SD)	16	0.29	6	Left
2.0	47.2 (-2 SD)	11	0.23	7	Left
2.0	50.7 (-1 SD)	13	0.26	7	Left
2.5	*ND*	11		8	Left
2.5	*ND*	13		1a	Right
2.5	60.0 (0 SD)	13	0.21	13c	Right
3.0	*ND*	11		8	Right
3.0	*ND*	12		1c	Right
3.0	*ND*	13		2a	Right
3.0	49.7 (-2 SD)	10	0.20	5c	Right
3.0	*ND*	12		10	Right
3.0	*ND*	13		1a	Left
3.0	59.0 (0 SD)	13	0.22	11	Left
3.5	*ND*	12		1b	Right
4.0	57.0 (0 SD)	12	0.20	9	Left
4.5	54.0 (0 SD)	8	0.15	11	Right
5.0	56.0 (0 SD)	8	0.14	9	Right
5.0	*ND*	6		3a	Left
5.0	45.0 (-2.5 SD)	11	0.24	4	Left
6.0	50.0 (-2 SD)	6	0.12	3a	Right
6.0	*ND*	6		1b	Left
7.0	*ND*	7		1c	Left
7.0	*ND*	10.2		10	Left
13.0	25.0 (-4 SD)	4	0.16	5a	Left
14.0	43.0 (-1 SD)	4.5	0.10	5b	Right
14.0	33.0 (-3 SD)	4.5	0.14	5b	Left
18.0	28.5 (-3 SD)	4	0.14	5a	Right

*SD* Standard deviation, *SD¥* Standard deviation of Distal Motor latency, *MNCV*_*p,d*_ Motor Nerve Conduction Velocity; distal, proximal, *ND* not done, *Left* Left arm, *Right* Right arm

a: 1st evaluation; b: 2nd evaluation; c: 3rd evaluation

## Discussion

This retrospective study of 13 children with MPS highlights the advantages of early ENG evaluation to detect CTS, although the low number of patients appears as a limitation of the study.

ENG anomalies appeared as early as 3 years and 7 months in the youngest child, whereas the average age of clinical symptoms was 6 years in our series, corroborating what has been previously reported [4]. ENG anomalies preceded clinical signs of CTS which were reported on average 44.6 months after the diagnosis of MPS in four of our patients. Thomas et al. [24] note that CTS is commonly reported during adolescence in attenuated MPS I. Given the much earlier onset of ENG anomalies, CTS diagnosis during adolescence is too late [8, 10, 15, 25].

Ulnar ENG evaluation showed no compression in the Guyon canal in any of the children. The integrity of median and ulnar nerves in the forearm, furthermore, normal tibial and fibular nerve conduction evaluations demonstrating absence of Tarsal Canal Syndrome, confirm absence of diffuse neuropathy. Therefore, we present findings of true median nerve compression with damage in the distal portion of the nerve, initially on the sensory pathway then becoming motor-sensory. This anomaly presented bilaterally, however the degree of severity was often asymmetrical. These findings are in accordance with classic ENG responses seen in adults with CTS [26].

Using Steven’s severity grade [23], 15 of the exams showed severe compression, 10 moderate and 8 minor.

Prolonged sensory and motor LDs are the hallmark of distal demyelination [26]. A prolonged LD (greater than 2SD) followed by diminished amplitudes, either sensory or motor, indicates a more pronounced stage of compression and is reflective of axonal distress [27]. We noted an interesting relationship between distal MLD, MNCV and ITL values (MNCVd / MNCVp). This ratio decreases as conduction time increases across the carpal tunnel, highlighting the slowing of distal conduction as compared to proximal [28]. In this study we have demonstrated that below a threshold of 14 m/s MNCV_d_ and ILT of 0.26 there is a true distal conduction slowdown. Threrefore it is important to study MNCV_p_ and MNCV_d_ bilaterally, even if this means a doubling of examination time. Due to lack of longitudinal data, we could not confirm a correlation between severity and age as is noted in the literature [29]. However, cross-sectionally we note that anomalies of the motor and sensory LD, amplitude and velocities are more pronounced in older age groups.

In our study, two of the children had diminished distal amplitudes in isolation; the amplitude measured at the elbow was normal. Sensory deterioration can remain severe from an electrical standpoint, as shown by the absence of sensory potentials. Infiltration at the wrist most likely renders measuring sensory responses difficult, causing decreased amplitudes without compression or axonal lesions [6, 7].

ERT is known to improve walking ability and respiration and enhances quality of life [30], and has been available in Europe for MPS I since 2003 and for MPS II since 2007 [14, 30]. However, there has been no observed correlation between ERT and improvements in CTS; many children have required surgical intervention despite ERT treatment [13, 15]. In our study, two children received ERT without surgical intervention, in one the child was very young (18 months) and was clinically asymptomatic and showed no electrical anomalies. The second child developed CTS despite four years of ERT.

Eight of the children in our series underwent a surgical intervention. The clinical follow-up in all these children was favorable, with increased comfort, absence of pain, increased joint flexibility, and consequent improvement in hand functionality. Despite the clinical improvements, two of the patients continued to show severe median, and essentially sensory nerve conduction abnormalities on ENG evaluations post-ERT and post-surgical treatment, most likely reflecting persistent technical challenges in obtaining results after infiltration [6, 7]. Five of the children had an ENG evaluation follow-up post-surgery, results of which showed marginal changes. However, three of these children had had ENG evaluations prior to surgery, and comparison of the pre- and post-surgical ENG evaluations showed marked improvements [8].

## Conclusions

In conclusion, in children with severe or attenuated MPS, the diagnosis of median nerve damage remains a challenge, especially since clinical symptoms are often non-specific, subtle or latent. Nonetheless, all patients with median nerve compression present with abnormal ENG recordings even in absence of clinical symptoms. ENG constitutes a powerful technique for detecting the initial signs of median nerve compression before the onset of symptoms by which time decompression surgery is less effective and there is a risk of axonal damage. To prevent or minimize irreversible sequelae of median nerve compression, we suggest annual electroneurography testing for mucopolysaccharidoses patients starting as early as 3 years of age, including both motor and sensory nerve pathways, on median and, in reference to the ulnar nerves, bilaterally at the wrist and the elbow. Furthermore, it is critical to calculate the ITL as it represents a sensitive index of distal velocity and serves to confirm any slight slowing in distal nerve conduction, given that the patient serves as his own control. We suggest using the threshold algorithm (ITL > 0.26) as a quick and simple means of detecting median nerve distal compression early on.

Timely surgical intervention can greatly improve the overall function and quality of life of these patients. It should be noted that some patients have no or minimal benefit following surgery and enzyme therapy and that carpal tunnel syndrome may occur despite enzyme replacement therapy: continued monitoring is therefore essential.

## Abbreviations

CMAP_d,p_: Compound Muscle Action Potential; distal, proximal
ENG: Electroneurography
ITL: Index of Terminal Latency
LLN: Lower limit of normal
MDL: Motor Distal Latency
MNCV_d_,_p_: Motor Nerve Conduction Velocity; distal, proximal
NCS: Nerve Conduction Study
SDL: Sensory Distal Latency
SEPA_d,p_: Sensory Evoked Potential Amplitude; distal, proximal
SNCV_d_,_p_: Sensory Nerve Conduction Velocity; distal, proximal

## References

[1] Albano LM, Sugayama SS, Bertola DR, Andrade CE, Utagawa CY, Puppi F, Nader HB, Toma L, Coelho J, Leistner S (2000). Clinical and laboratorial study of 19 cases of mucopolysaccharidoses. Rev Hosp Clin Fac Med Sao Paulo.

[2] Baehner F, Schmiedeskamp C, Krummenauer F, Miebach E, Bajbouj M, Whybra C, Kohlschütter A, Kampmann C, Beck M (2005). Cumulative incidence rates of the mucopolysaccharidoses in Germany. J Inherit Metab Dis.

[3] Martin R, Beck M, Eng C, Giugliani R, Harmatz P, Muñoz V, Muenzer J (2008). Recognition and diagnosis of mucopolysaccharidosis II (hunter syndrome). Pediatrics.

[4] Yuen A, Dowling G, Johnstone B, Kornberg A, Coombs C (2007). Carpal tunnel syndrome in children with mucopolysaccharidoses. J Child Neurol.

[5] White KK (2011). Orthopaedic aspects of mucopolysaccharidoses. Rheumatology.

[6] Wraith JE, Alani SM (1990). Carpal tunnel syndrome in the mucopolysaccharidoses and related disorders. Arch Dis Child.

[7] Al Sawaf S, Mayatepek E, Hoffmann B (2008). Neurologic findings in hunter disease: pathology and possible therapeutic effects reviewed. J Inherit Metab Dis.

[8] Haddad FS, Jones DH, Vellodi A, Kane N, Pitt MC (1997). Carpal tunnel syndrome in the mucopolysaccharidoses and mucolipidoses. J Bone Joint Surg Br.

[9] Norman-Taylor F, Fixsen JA, Sharrard WJ (1995). Hunter’s syndrome as a cause of childhood carpal tunnel syndrome: a report of three cases. J Pediatr Orthop B.

[10] Van Heest AE, House J, Krivit W, Walker K (1998). Surgical treatment of carpal tunnel syndrome and trigger digits in children with mucopolysaccharide storage disorders. J Hand Surg Am.

[11] Miebach E (2009). Management of infusion-related reactions to enzyme replacement therapy in a cohort of patients with mucopolysaccharidosis disorders. Int J Clin Pharmacol Ther.

[12] Muenzer J (2014). Early initiation of enzyme replacement therapy for the mucopolysaccharidoses. Mol Genet Metab.

[13] Parini M, Rigoldi M, Tedesco L, Boffi L, Brambilla A, Bertoletti S, Boncimino A, Del Longo A, De Lorenzo P, Gaini R (2015). Enzymatic replacement therapy for hunter disease: up to 9 years experience with 17 patients. Mol Genet Metab Rep.

[14] Scarpa M, Almássy Z, Beck M, Bodamer O, Bruce IA, De Meirleir L, Guffon N, Guillén-Navarro E, Hensman P, Jones S (2011). Mucopolysaccharidosis type II: European recommendations for the diagnosis and multidisciplinary management of a rare disease. Orphanet J Rare Dis.

[15] Khanna G, Van Heest AE, Agel J, Bjoraker K, Grewal S, Abel S, Krivit W, Peters C, Orchard PJ (2007). Analysis of factors affecting development of carpal tunnel syndrome in patients with hurler syndrome after hematopoietic cell transplantation. Bone Marrow Transplant.

[16] Bahadir C, Kurtulus D, Cihandide E (2009). Mucopolysaccharidosis type-IS presenting with onset of carpal tunnel syndrome at adolescence. J Clin Rheumatol.

[17] Kanaan N, Sawaya RA (2001). Carpal tunnel syndrome: modern diagnostic and management techniques. Br J Gen Pract.

[18] Werner RA, Andary M (2002). Carpal tunnel syndrome: pathophysiology and clinical neurophysiology. Clin Neurophysiol.

[19] Valayannopoulos V, Wijburg FA (2011). Therapy for the mucopolysaccharidoses. Rheumatology.

[20] Neufeld E, Muenzer J, Scriver CR, Beaudet AL, Sly WS, Valle D, Childs B, Kinzler KW, Vogelstein B (2001). The mucopolysaccharidoses. The metabolic and molecular basis of inherited disease. Volume III.

[21] Fournier E (1998). Examen électromyographique et étude de la conduction nerveuse-sémiologie électrophysiologique.

[22] Raimbault J (1988). Les conductions nerveuses chez l'enfant normal.

[23] Stevens JC (1997). AAEM minimonograph # 26: the electrodiagnosis of carpal tunnel syndrome. American Association of Electrodiagnostic Medicine. Muscle Nerve.

[24] Thomas JA, Beck M, Clarke JT, Cox GF (2010). Childhood onset of Scheie syndrome, the attenuated form of mucopolysaccharidosis I. J Inherit Metab Dis.

[25] Holt JB, Van Heest AE, Shah AS (2013). Hand disorders in children with mucopolysaccharide storage diseases. J Hand Surg Am..

[26] Davis L, Vedanarayanan VV (2014). Carpal tunnel syndrome in children. Pediatr Neurol.

[27] Seror P (1996). The axonal carpal tunnel syndrome. Electroencephalogr Clin Neurophysiol.

[28] Fournier E (2008). Examen électromyographique : Sémiologie électrophysiologique des nerfs et des muscles.

[29] Kwon J-Y, Ko K, Sohn YB, Kim SJ, Park SW, Kim SH, Cho SY, Jin DK (2011). High prevalence of carpal tunnel syndrome in children with mucopolysaccharidosis type II (hunter syndrome). Am J Med Genet A.

[30] Muenzer J, Beck M, Eng CM, Giugliani R, Harmatz P, Martin R, Ramaswami U, Vellodi A, Wraith JE, Cleary M (2011). Long-term, open-labeled extension study of idursulfase in the treatment of hunter syndrome. Genet Med.

